# Diagnostic Applications of Saliva in Dentistry

**DOI:** 10.5005/jp-journals-10005-1012

**Published:** 2009-12-26

**Authors:** Prabhakar AR, Akanksha Gulati, Deepak Mehta, S Sugandhan

**Affiliations:** 1 Professor and Head, Department of Pedodontics and Preventive Dentistry, Bapuji Dental College and Hospital, Davangere 577004, Karnataka, India; 2 Postgraduate Student, Department of Pedodontics and Preventive Dentistry, Bapuji Dental College and Hospital Davangere-577004, Karnataka, India; 3 Postgraduate Student, Department of Pedodontics and Preventive Dentistry, Bapuji Dental College and Hospital, Davangere 577004, Karnataka, India; 4 Professor, Department of Pedodontics and Preventive Dentistry, Bapuji Dental College and Hospital, Davangere-577004 Karnataka, India

**Keywords:** Saliva diagnosis, caries, caries activity tests.

## Abstract

*Background:* The use of saliva to identify individuals with disease and to follow the progress of the affected individual has attracted the attention of numerous investigators. Its noninvasive method of collection, simplicity, and cost effectiveness make it a useful tool not only to the general practitioner but also to the pediatric dentist.

*Aim:* The aim of this paper is to provide the clinician with a comprehensive review of the diagnostic uses of saliva in dentistry.

## INTRODUCTION

The analysis of blood, the most frequent specimen in clinical chemistry, has two purposes: first, to identify individuals with disease and second, to follow the progress of the affected individual under medical treatment. A similar use has been envisioned for salivary secretions. The diagnostic use of saliva has attracted the attention of numerous investigators because of the noninvasive nature and relative simplicity of collection. The clinical applications of saliva range from the forensic field to drug monitoring and diagnosis of systemic and local conditions.^1^

Saliva is not one of the popular bodily fluids. It lacks the drama of blood, the sincerity of sweat and the emotional appeal of tears. Despite the absence of charisma, a growing number of internists, pediatricians, pharmacologists, clinical and forensic pathologists, endocrinologists, immunologists, psychologists and dentists are finding that saliva provides an easily available, noninvasive diagnostic medium for a rapidly widening range of diseases and clinical situations.^2^

The salivary glands are composed of specialized epithelial cells, and their structure can be divided into two specific regions: The acinar and the ductal regions. The acinar region is where fluid is generated and most of the protein synthesis and secretion takes place. The autonomic nervous system (sympathetic and parasympathetic) controls the salivary secretion. The signaling mechanism involves the binding of neurotransmitter (primarily acetylcholine and norepinephrine) to plasma membrane receptors and signal transduction via guanine nucleotide-binding regulatory proteins (G-proteins) and activation of intracellular calcium signaling mechanisms.^3^,^4^

Broad overview of the diagnostic applications of saliva:

- Systemic diseases
  – Hereditary
  – Autoimmune
  – Malignancy
  – Infectious
- Viral diseases
- Drug monitoring
- Monitoring of hormone level
- Diagnosis of oral disease with relevance for systemic diseases
- Caries activity testing
- Forensic odontology

There are several ways by which serum constituents that are not part of the normal salivary constituents (*i.e*., drugs and hormones) can reach saliva. Within the salivary glands, transfer mechanisms include intracellular and extracellular routes. The most common intracellular route is passive diffusion, although active transport has also been reported. Ultrafiltration, which occurs through the tight junctions between the cells, is the most common extracellular route. Serum constituents are also found in whole saliva as a result of GCF outflow. Depending on the degree of inflammation in the gingiva, GCF is either a serum transudate or, more commonly, an inflammatory exudate that contains serum constituents.^3^,^5^

Salivary diagnostics is a developing field with the growing appreciation of saliva as a mirror of the body reflecting:

1. Tissue fluid levels of natural substances and a large variety of molecules introduced for therapeutic, dependency or recreational purposes.
2. Emotional status from high anxiety to low-down blues, from mania to depression.
3. Hormonal status.
4. Immunological status and responsiveness.
5. Neurological effects.
6. Nutritional and metabolic influences.^6^

There are compelling reasons to use saliva as a diagnostic fluid to monitor health and disease. It meets the demands for inexpensive, noninvasive, and easy to use diagnostic methods. As a clinical tool, saliva has many advantages over serum. It is easy to collect, store, ship, and it can be obtained at low cost in sufficient quantities for analysis. For patients, the noninvasive collection techniques dramatically reduce anxiety and discomfort, and simplify procurement of repeated samples for longitudinal monitoring over time. This is an added advantage for its use in pediatric patients. For professionals, saliva collection is safer than blood tests, which could expose health care workers to HIV or hepatitis virus. Saliva is also a lot easier to handle for diagnostic procedures, since it does not clot, thus lessening the manipulations required. Saliva based diagnostics is therefore more accessible, accurate, less expensive, and presents less risk to the patient than current methodologies.^7^

The purpose of this article is to review the diagnostic applications of saliva in dentistry.

## SALIVARY PARAMETERS OF RELEVANCE FOR ASSESSING CARIES ACTIVITY IN INDIVIDUALS AND POPULATIONS

In clinical practice, measurement of saliva (sialometry) is particularly indicated:

1. As part of the initial examination of a new patient to be treated for dental caries.
2. During evaluation of preventive and restorative treatment of dental caries, to assess how the overall treatment has affected oral health.
3. In elderly patients who take regular medication, and/or have exposed root surfaces.
4. As part of the investigative procedures for suspected hyposalivation associated with, for example, regular use of medicines with systemic depressive effects on salivary flow rates, Sjogrens syndrome and other diseases, or irradiation to the head and neck region.^8^

### Salivary Flow Rate

Probably the most important caries-preventive functions of saliva are the flushing and neutralizing effects, commonly known as salivary clearance. In general, the higher the flow rate, the faster the clearance, and the higher the buffer capacity.^9^

Salivary flow from major and minor glands is controlled by both parasympathetic and sympathetic stimuli of various types and, depending on the nature of the stimulus, this also affects the composition of saliva. From the clinical point of view, this difference in stimuli is of minor relevance since both the amount of fluid and the concentration and nature of salivary proteins are important in protection against microbial diseases, such as dental caries. The actual antimicrobial activity of saliva resides mainly in the protein fraction whereas the water/electrolyte fraction is more important in the clearance process.

For a clinician, the saliva in fact means "whole saliva" which is the fluid present in the mouth and comprises not only pure secretions from the major and minor salivary glands but also gingival exudate, microorganisms and their products, epithelial cells, food remnants and also to some extent nasal exudate. The flow of this whole saliva is of clinical relevance for the susceptibility and activity of dental caries. However, it must be emphasized that no linear association exists between salivary flow rate and caries activity but rather it is a question of "threshold effect".

From the cariological perspective, crossing the individual "threshold level" rapidly increases caries activity if preventive measures are not adequate. The most common reasons for the decline of salivary output below the threshold level are:

- Medication (antidepressants, diuretics, antihistamines, narcotics, beta adrenergic agonists)
- Irradiation to the head and neck area
- Autoimmune diseases (rheumatoid arthritis, Sjogren’s syndrome)
- Menopause
- Anorexia nervosa, malnutrition, frequent long-lasting fasting
- Labile type I diabetes mellitus.

Since many recent sialoepidemiological studies have indicated that hyposalivation and/or xerostomia are relatively common, exceeding 20% already among young adults, salivary flow is without doubt the most important single saliva factor controlling the development of dental caries. Medication, which is increasingly common even among adolescents, is the single most important factor controlling the development of dental caries.^10^

Stimulated salivary flow rates of less than 0.7 ml/min are regarded as a threshold for considerably increased risk of further caries development. As the most important clinical variable of saliva affecting susceptibility to dental caries, simple quantitative assessment of stimulated whole saliva should be a routine dental procedure.^8^

### Regulation of Salivary pH^10^

The association between low caries levels and high salivary buffering has been demonstrated convincingly.^11^ The human mouth is quite frequently exposed to components whose pH differs from saliva’s normal pH (6.5-7.5). These components may cause damage to teeth (erosion) or mucosal surfaces. Buffering agents in saliva, however, try to bring the pH back to the normal range as fast as possible. In resting saliva the major buffering agent is inorganic phosphate and in stimulated saliva carbonic acid/bicarbonate system. At very low pH (4-4.5) salivary proteins also display some buffer action.

Clinical studies have shown that on a population level a negative association between buffering and dental caries exists but salivary buffer capacity tests alone have only a weak association with caries activity or outcome of future caries. It must be stressed that the decisive processes in caries attack occurs within dental plaque or even beneath the outer surface of enamel where the action of salivary buffering agents is minimal or negligible. Therefore the buffer effect, as measured from whole saliva, is obviously more reliable as a diagnostic test in assessing the predictive power of dental erosion than that of dental caries.

### Antimicrobial Agents of Saliva and Caries Activity^10^

A number of antimicrobial agents have been identified in human saliva. These are usually divided into nonimmune and immune (immunoglobulin) factors. A vast number of reports document how these factors alone or in combination affect cariogenic microorganisms, in particular mutans Streptococci.

The major salivary antimicrobial agents are:

- Secretory IgA
- Lysozyme
- Lactoferrin
- Salivary peroxidase/myeloperoxidase
- Hypothiocyanite
- Agglutinins
- Histamins.

It is highly unlikely that any single antimicrobial agent of human saliva could have a strong, perhaps even predictive, association with caries susceptibility or activity. This is due to the following reasons:^10^

1. Antimicrobial agents are dependent on salivary flow rate in an individual manner.
2. Depending on the simultaneous extent of gingival inflammation, the crevicular exudate, especially lysed neutrophils, provides antimicrobial agents (such as lysozyme, lactoferrin, myeloperoxidase, immunoglobulins) into the whole saliva, irrespective of the caries status.
3. The measurement of total secretory IgA of saliva is too crude with respect to caries prevalence or incidence and specific antimutans Streptococci IgAs vary according to the time of infection.
4. Among patients with humoral immunodeficiency the nonimmune salivary antimicrobial proteins are normal or even compensate for the lack of antibodies.
5. Intra- and interindividual variation is large and agedependent. Furthermore, many antimicrobial salivary proteins are susceptible to proteolysis by plaque bacterial enzymes.
6. Salivary antimicrobial proteins (both immune and nonimmune) interact in many ways by either enhancing or inhibiting other proteins’ functions.
7. A single antimicrobial agent may interact with cariogenic bacteria in many ways, e.g. by either supporting or inhibiting bacterial adhesion to saliva coated apatite (for e.g. secretory IgA and proline-rich proteins).

The above observations, together with the fact that so far longitudinal multivariate analyses of salivary antimicrobial agents in relation to initiation of caries are virtually lacking, offer at the moment no clearcut diagnostic value either on an individual or on a population level.

### Fluoride

Fluoride is one of the major constituents of saliva to control caries activity. The fluoride concentration in saliva is usually low, in the range of 0.4-2.6 µm but increases in fluoridated areas. Salivary fluoride will diffuse into the dental plaque where some of the fluoride will be trapped as calcium fluoride. Plaque and salivary fluoride contribute significantly to the remineralization process but it should be kept in mind that there is a constant clearance (elimination) of fluoride from the mouth. This clearance is rapid during the first 20- 40 minutes but then slowly levels off depending on the salivary flow rate and the volumes of saliva in the mouth before and after swallowing. Although the fluoride clearance influences the acid production in the plaque as well as caries scores, the diagnostic value of salivary fluoride measurement at both individual and population level is as yet questionable.

Although strong evidence exists that many salivary nonmicrobial parameters affect the caries process, on an individual or population basis they offer only little diagnostic or predictive value. Salivary flow rate, buffer effect and perhaps also the *in vivo* concentrations of some salivary constituents such as fluoride, hypothiocyanite and agglutinins (possibly including IgA) seem to be more important than the others in assessing caries susceptibility and/or activity.^10^

Studies show that salivary parameters such as salivary flow rate, salivary viscosity, salivary pH and salivary buffering capacity were lower in subjects with high dental caries. Hence it is recommended that salivary testing be a part of routine diagnosis when treating patients with high risk of dental caries.^12^

## SALIVA BASED CARIES ACTIVITY TESTS

Evaluating the causative factors in saliva of individual’s at risk to dental caries can pave the way to make recommendations that will cater specifically to the individual’s needs. Many benefits exist for both patients and dentists by introducing saliva testing as part of practice philosophy. The practice can benefit from enhanced diagnostics, early detection of problems, improved patient communication and motivation and an increased dental awareness for patients.^12^

Caries activity tests have been used in dental research for many years, and some tests have been adapted for routine use in the dental office. There is no ideal test in existence at the present time, although caries activity tests are a valuable adjunct for patient motivation in a plaque control program. Saliva serves as a major component of most caries activity tests, and aids in the categorization of patients into high, medium and low caries activity.^13^

Some of the important caries activity tests are as follows.

## LACTOBACILLUS COLONY COUNT TEST

This test was first introduced by Hadley in 1933. It estimates the number of acidogenic and aciduric bacteria in the patient’s saliva by counting the number of colonies appearing on tomato peptone agar plates (pH 5.0) after inoculation with a sample of saliva. The total number of colonies on this medium reflects the proportion of the aciduric flora in the saliva. The lactobacillus plate count is one of the oldest caries activity test. It helps categorize the patient into little or none, slight, moderate and marked caries activity groups via the saliva. A highly practical and greatly simplified method of estimating lactobacilli is now available - Dentocult, orion diagnostica.

The lactobacilli count test can be used for planning recall intervals, as an educational aid and monitoring mechanism in dietary counseling, for treatment planning (for example, a steady high count contraindicates orthodontics, bridges, implants), and for identification for a medically compromised patient (for example, a steady high count occurs in diabetes mellitus).^14^

## SNYDER TEST^13^,^15^

At present the best known test is the Snyder’s test.^16^ The Snyder test measures the rapidity of acid formation when a sample of stimulated saliva is inoculated into glucose agar adjusted to pH 4.7 to 5 and with bromocresol green as color indicator. Indirectly, the test is also a measure of acidogenic and aciduric bacteria. It categorizes the patient into limited, definite and marked caries activity groups. The Snyder test is simple, takes 24-48 hours, and requires only simple equipment; some training is needed and the cost is moderate. It utilizes a salivary sample which is easy to obtain.

This test meets some of the ‘ideal test’ characteristics. Snyder and others have found a high correlation between the Snyder acid production test and the lactobacillus plate count test. Also, Snyder and others have found a high correlation between clinical caries activity and positive Snyder test results on a group basis. The best agreement was between a negative Snyder test and the absence of caries activity.

Primary interest in the lactobacilli and mutans Streptococci in caries activity tests stem from the fact that caries-conductive conditions are associated with elevated levels of these organisms in saliva. Strong evidence also indicates that the association of lactobacilli and mutans Streptococci with caries development is linked directly to carbohydrate consumption which, in turn, is one of the indispensible factors in caries development.^16^

Although these salivary microbial tests are not very useful for determining future individual caries activity, there are some legitimate indications for salivary microbial tests.^17^ Pediatricians should be convinced to use these tests regularly on children under the age of four because they have a greater chance to see infants before damage occurs. The success of chemoprophylactic measures such as chlorhexidine varnish can be accurately monitored by these tests. Compliance with dietary recommendations can also be controlled. Salivary microbial tests are also of value in the surveillance of oligosialic and xerostomic patients. When fixed bands are used by orthodontists these tests may provide precautionary signals. And last the cooperation and motivation of a cariesactive individual towards preventive measures may be increased if the results of such tests are shown and their clinical significance explained to the individual.

## REDUCTASE TEST^18^,^19^

The test measures the rate at which an indicator molecule, diazoresorcinol, changes from blue to red to colorless or leukoform on reduction by the mixed salivary flora. Rapp claims the test "measures the activity of a single enzyme, reductase. This enzyme is involved in some very definite and limiting reactions in the formation of products dangerous to the tooth surface."

Rapp has claimed a good correlation of the results of this test with clinical caries experience. Other investigators concluded that this test did not give accurate results and was not of diagnostic value, but a correlation between reductase activity and the numbers of salivary anaerobes has been reported. Caries-free adults exhibit low or negative scores on the reductase test. It has been proposed that this test is rather a measure of the oral hygiene status of the individual. Test results vary with time after food intake and after brushing.

## BUFFER CAPACITY TEST

Buffer capacity can be quantitated using either a pH meter or color indicators. The test measures the number of milliliters of acid required to lower the pH of saliva through an arbitrary pH interval, such as from pH 7.0 to 6.0, or the amount of acid or base necessary to bring color indicators to their end points.

There is an inverse relationship between buffering capacity of saliva and caries activity. The saliva of individuals whose mouths contain a considerable number of carious lesions frequently has a lower acid-buffering capacity than the saliva of those who are relatively caries-free. This test, however, does not correlate adequately with caries activity.

## FOSDICK CALCIUM DISSOLUTION TEST

The test measures the milligrams of powdered enamel dissolved in 4 hours by acid formed when the patient’s saliva is mixed with glucose and powdered enamel. In limited studies, the correlation is reported to be good. The time required is 4 hours. However, this test is not simple, the equipment is complex, personnel must be trained, and the cost is high.

## *S. MUTANS* ADHERENCE METHOD

The test categorizes salivary samples based on the ability of *S. mutans* to adhere to glass surfaces when grown in sucrose-containing broth. This method is potentially useful for handling many samples in preventive practice and epidemiological studies because of its simplicity.

## *S. MUTANS* DIP-SLIDE METHODS

These tests (*Dentocult SM, Orion Diagnostica; Cariesscreen SM, Apo diagnostics*) classify salivary samples according to estimates of *S mutans* colonies growing on modified mitis-salivarius agar.

Studies show that the presence of mutans Streptococci both in plaque or saliva of young caries-free children of age 2-5 years is associated with a considerable increase of caries risk. However, recommendations for the general use of the *Streptococcus* mutans test as a risk assessment tool in preschool children cannot be justified yet due to lack of well-designed studies.^20^

Some indications for these tests as predictors of caries risk in particular ages groups have been described as follows. With reference to infants and preschoolers, the caregiver can be tested instead of the child, since the infective nature of caries has been well-demonstrated with the ready transfer of putative microorganisms from this individual to the child. Caregivers considered to provide a significant risk of infecting a child can then receive appropriate restorative and preventive care, of benefit to both themselves and the child.

For school age children, the caries risk for individuals and for groups can be determined, with particular reference to planning lengths of recall periods and, in the public sector, planning the allocation of resources. For adolescents, the tests can be of predictive value before and during orthodontic treatment. With reference to adults, unexplained new high caries rates can be studied with these tests and response to recommended alterations in dietary habits or oral hygiene can be monitored. And finally in the elderly, increases in caries rates associated with lifestyle changes such as systemic disease, medications, or social change, can be studied and monitored in response to the institution of oral health measures.^14^

*Tests for fungi*: Salivary yeasts can also be assayed and raised values can give indication of oral conditions such as denture stomatitis, oral infections following the use of broad spectrum antibiotics, low or altered immune responses, Sjogren’s syndrome and age. They can also be used to monitor the efficacy of antifungal therapy, and to assess an aciduric environment, which fungi use and which also favors caries. The presence of an oral yeast infection may reflect general host response and indicate a medically compromised patient.^14^

## PERIODONTAL DIAGNOSIS

It has long been realized that a rapid and simple diagnostic test that can provide a reliable evaluation of periodontal disease and identify patients at risk for active disease would be of value to both clinicians and patients. Saliva may offer the basis for a patient specific diagnostic test for periodontitis.^21^ The use of saliva for periodontal diagnosis has been the subject of considerable research activity, and proposed markers for disease include proteins of host origin (i.e. enzymes, immunoglobins), phenotypic markers (epithelial keratins), host cells, hormones, bacteria and bacterial products, volatile compounds and ions.^22^

### Enzymes as Markers

In a study by Nakamura and Slots, 1983, enzyme activity in mixed whole saliva and parotid saliva was examined in individuals with a healthy periodontium, and compared with individuals with adult periodontitis (AP) and localized juvenile periodontitis (LJP). Mixed whole saliva from AP patients demonstrated the highest enzyme activity, while healthy controls showed the lowest. Their data suggested that oral microorganisms contributed to the enzyme pool in saliva. Zambon et al, 1985, also proposed that the effectiveness of periodontal treatment might be monitored by changes in levels of specific bacterial enzymes in whole saliva.

Children with Down’s syndrome have an increased prevalence and severity of periodontal disease. Activity of enzyme salivary collagenase was found to be higher in these children.

(Other enzymes studied were gelatinase, lysozyme, elastase).

### Immunoglobulins

Changes in the concentration of immunoglobulins in saliva following treatment were examined by Reiff, 1984. A decrease in the salivary levels of both IgA and IgG was observed after treatment. The reduction in IgA and IgG was more consistent in patients with less severe periodontitis. For these patients the immunoglobulin levels in saliva proved to be a better indicator of the local response than the serum titer.

### Bacteria

Salivary levels of periodontal pathogens were found to vary with periodontal status and as a result of treatment. Salivary levels of *A. actinomycetemcomitans, P. gingivalis, P. intermedia, Campylobacter rectus,* and *Peptostreptococcus micros* were determined by bacterial culture and related to subjects with varying degrees of periodontitis. The salivary levels of the periodontal pathogens reflected the periodontal status of the patient. Furthermore in patients that received mechanical debridement and adjunctive systemic metronidazole, periodontal treatment eradicated or significantly reduced the levels of the periodontal pathogens in saliva.^23^

Other markers also studied are ions (e.g. calcium), hormones (e.g. cortisol), inflammatory cells, epithelial keratins, etc.

## CONCLUSION

The ability to monitor health status, disease onset and progression, and treatment outcome through noninvasive means is the most desirable goal in health-care promotion and delivery. There are three prerequisites for this goal to be realised: specific biomarkers associated with a health or disease state, a noninvasive approach to detect and monitor these biomarkers, and the technologies to discriminate between and among biomarkers.

The ability to utilize saliva to monitor the health and disease state of an individual is a highly desirable goal for health promotion and health-care research. However, saliva diagnostics is a ‘late bloomer’, since only recently has there been a growing appreciation of saliva as a mirror of the body which can reflect virtually the entire spectrum of normal and disease states.^7^
